# Simplifying and optimising management of acute malnutrition in children aged 6 to 59 months: study protocol for a 3 arms community-based individually randomised controlled trial in decentralised Niger

**DOI:** 10.1186/s13063-021-05955-6

**Published:** 2022-01-28

**Authors:** Maguy Daures, Jérémie Hien, Kevin Phelan, Harouna Boubacar, Sanoussi Atté, Mahamadou Aboubacar, Ahmad A. G. M. Aly, Baweye Mayoum, Jean-Claude Azani, Jean-Jacques Koffi, Benjamin Séri, Aurélie Beuscart, Delphine Gabillard, Victoire Hubert, Cécile Cazes, Moumouni Kinda, Xavier Anglaret, Suvi Kangas, Susan Shepherd, Renaud Becquet

**Affiliations:** 1 Fench National Institute for Health and Medical Research (Inserm), French National Research Institute for Sustainable Development (IRD), Bordeaux Population Health Research Center, Team IDLIC, UMR 1219, University of Bordeaux, 146 rue Léo Saignat, 33076 Bordeaux, France; 2 The Alliance for International Medical Action (ALIMA), Zinder, Niger; 3 The Alliance for International Medical Action (ALIMA), Paris, France; 4 Nutrition Directorate, Ministry of Health, Niamey, Niger; 5 Commission for the Initiative “les Nigériens Nourrissent les Nigériens” (HC3N), Niamey, Niger; 6 The Alliance for International Medical Action (ALIMA), 15 rue des immeubles industriels, 75011 Dakar, Senegal; 7 PACCI Research Programme, University Hospital of Treichville, Abidjan, Côte d’Ivoire; 8 International Rescue Committee (IRC), Dakar, Senegal

## Abstract

**Background:**

Simplified approaches of acute malnutrition (AM) treatment have been conducted over the past 5 years intending to unify processes and increase coverage among children aged 6 to 59 months without medical complication. The Optimsing treatment for Acute Malnutrition (OptiMA) and the Combined Protocol for Acute Malnutrition Study (ComPAS) are mid-upper arm circumference (MUAC)-based approaches treating children with MUAC < 125 mm or oedema with one sole product—ready-to-use therapeutic food—at a gradually tapered doses. This trial aims to compare the OptiMA and ComPAS strategies to the standard nutritional protocol of Niger assessed by a favourable outcome in the treatment of uncomplicated AM at 6 months post-randomisation and in terms of recovery rate after treatment of uncomplicated SAM (WHZ < − 3 or MUAC < 115mm or oedema) and among the most vulnerable children (MUAC < 115mm or oedema).

**Methods:**

A non-inferiority individually randomised controlled clinical trial was conducted at the primary health centres level and in the community in the Zinder region in Niger in March 2021. Participants are children aged 6–59 months attending outpatient health centres with MUAC < 125mm or oedema without medical complications. All participants are followed for 6 months. Simplified strategies propose a gradual reduction of RUTF according to MUAC and weight in OptiMA and MUAC only in ComPAS. Favourable outcome is compositely defined at 6 months post-inclusion as being alive, not acutely malnourished by the definition applied at inclusion and without any additional episode of AM throughout the 6-month observation period. Recovery is defined throughout the 6 months post-randomisation by a minimum of 4-week duration of treatment, an axillary temperature < 37.5°C, an absence of bipedal oedema and a MUAC ≥ 125 mm for two consecutive weeks. The sample size calculation required 567 children per arm for the main objective, 295 and 384 children per arm for the secondary objectives among SAM and MUAC < 115 mm children, respectively. Per-protocol and intention-to-treat analyses will be conducted for each outcome.

**Discussion:**

This trial is intending to generate much-needed evidence on various simplified and optimised AM treatment approaches and to participate in reaching a consensus on such nutrition protocols.

**Trial registration:**

ClinicalTrials.govNCT04698070. Registered on January 6, 2021

**Supplementary Information:**

The online version contains supplementary material available at 10.1186/s13063-021-05955-6.

## Introduction

Acute malnutrition (AM) affected an estimated 45.5 million children under 5 years of age in 2020, including 13.6 million suffering from severe acute malnutrition (SAM) [1], and was an underlying cause of 800,000 yearly deaths worldwide [2]. The coverage rate for community-based management of acute malnutrition (CMAM) programmes is low, with less than 25% of all children suffering from SAM being admitted to treatment, and an even lower proportion of children with moderate acute malnutrition (MAM) accessing support [3]. This low coverage is multi-factorial with the following two issues having been particularly underlined: first, the complexity of nutrition programming which uses different case definitions based on weight-for-height *Z*-score (WHZ) and/or middle-upper arm circumference (MUAC) and/or oedema; second, the quantity of ready-to-use therapeutic food (RUTF) given to children in current standard protocols are not optimum [4], which negatively impacts the actual cost of such programmes. In standard protocols, RUTF quantity prescribed in the first weeks of treatment is paradoxically often less important than what is given to children reaching recovery (MUAC > 125 and/or WHZ > − 2), because the weekly ration is determined by the child’s weight, which increases as the child recovers. There is a growing body of evidence suggesting that weight gain is highest in the first month of treatment and then plateaus [5]. There would therefore be no benefit of increased RUTF ration during the course of treatment, and progressive reduction of RUTF quantity seems to be a more rational use of this treatment.

In addition, SAM and MAM programmes are chronically underfunded with less than 40% of SAM cases treated globally in 2019 and as few as 16% of MAM cases reached by the World Food Program in 2017 [6, 7]. These two programmes are managed separately, with programmes overseen by different UN agencies, and using different protocols and products. An optimised allocation of resources is therefore urgently needed. To address these issues, simplified and optimised approaches have been conducted in Sub-Saharan Africa over the past 5 years intending to simplify processes and increase coverage among children aged 6 to 59 months without medical complications [8]. Protocol modifications are varied and still evolving, but a common approach using 1- only MUAC or oedema for admission, follow-up, and discharge, 2- a simplified definition of AM by MUAC < 125 mm or presence of bipedal oedema, and 3- a single product for treatment (RUTF) at different gradually reduced dosages has been evaluated in recent randomised controlled trials.

The first cluster-randomised controlled trial based on MUAC < 125mm or oedema was exploring the efficacy of an integrated MUAC-based SAM/MAM treatment protocol using RUTF only but at different doses for children < 115mm (175 kcal/kg/day) and 115 to 124 mm (75 kg/kcal/day) compared to the standard protocol in Sierra Leone in 2013 [9]. The results showed that the integrated programme had a reduced caseload of SAM, due to earlier treatment of children presenting as MAM, with a similar recovery rate (83–95% CI 81–85 vs. 79–95% CI 77–82) and higher coverage (71% vs. 55%, *p* = 0.0005).

The Optimsing treatment for Acute Malnutrition (OptiMA) strategy has been developed in a research consortium between humanitarians from the Alliance for International Medical Action (ALIMA) non-governmental organisation and scientists from the French National Institute for Health and Medical Research (Inserm). The OptiMA RUTF ration is calibrated to the child’s degree of wasting based on the combination of MUAC status and weight. Thus, more nutritional feeding is given to the most severely malnourished and then gradually reduced as the child’s MUAC increased. Children with MUAC < 115 mm or oedema receive 175 kcal/kg/day of RUTF. Children with MUAC 115–119 mm, either at admission or during the course of treatment, receive 125 kcal/kg/day of RUTF, and children with MUAC ≥ 120 mm receive 75 kcal/kg/day of RUTF (with a minimum of one sachet/day) until discharge from the programme. The OptiMA strategy was first implemented in a proof-of-concept single-arm trial in Burkina Faso in 2018 [10]. The results showed a recovery rate that exceeded SPHERE standards among children admitted with MUAC < 125mm (86.3%; 95% CI 85.4–87.2%) with an excellent health worker adherence to the new RUTF table. However, recovery among children admitted with a MUAC < 115 mm or oedema was poorer than anticipated (70.4%; 95% CI 67.5–73.5%). Then, an individually randomised non-inferiority controlled trial compared the OptiMA strategy to the national protocol was implemented in the Democratic Republic of the Congo (DRC) in 2019 [11]. The OptiMA protocol treated 29% more children with 20% less nutritional inputs compared to the standard protocol. A pilot non-comparative cohort study on 1111 acutely malnourished children per the OptiMA definition was implemented in 2019 in Niger and showed a similar result to the one observed in the above-mentioned study in Burkina Faso with a global recovery rate among children admitted with a MUAC < 125 mm that exceeded 80% ( [10] and Personal communication, 2020). However, recovery among the 290 children admitted with a MUAC < 115 mm remained low (57.7%), partly explained by a high proportion of children (34.1%) not reaching the exit criteria after 10 weeks of participation in the programme (Personal communication, 2020).

The Combined Protocol for Acute malnutrition Study (ComPAS) was developed by the International Rescue Committee (IRC) and consists in reducing the dosage according to the child’s MUAC during the outpatient follow-up and has the simplest dosing regimen ever tested: 2 RUTF sachets/day to children with MUAC < 115 mm or oedema and 1 RUTF sachet/day for children in the 115–124 mm MUAC category [12]. The main advantage of this strategy is that it can be implemented by community health workers, regardless of literacy level and without other anthropometric tools rather than a MUAC tape. Simplification and decentralisation of nutrition programming to the community level stand the best chance of increasing service coverage. A cluster-randomised controlled non-inferiority trial was implemented in Kenya and South Sudan in 2017 and showed that recovery in the ComPAS arm (76.3%) was non-inferior to that of the standard care arm (73.5%): risk difference of 0.03, 95% CI − 0.05 to 0.10, *p =* 0.52 [13]. The average amount of RUTF for SAM children was significantly lower under the ComPAS protocol (122 sachets) compared to the standard one (193 sachets) [13].

These simplified nutrition protocols deviate from existing normative guidance, and despite the MUAC-based common approach, there is currently a lack of consensus on these modifications. More evidence is needed prior to developing such simplified and optimised approaches at scale. Burning issues include which dosage is better to implement and what is the impact of such reduced regimens on recovery, especially among the most vulnerable children (MUAC < 115 mm).

This is exactly what this community-based non-inferiority trial proposes by comparing the OptiMA and ComPAS strategies to the current Niger protocol of AM treatment. We hypothesised that the OptiMA and ComPAS strategies would be as effective as the current Niger national protocol currently in use in children aged 6 to 59 months. This hypothesis will be assessed by reaching a favourable outcome in the treatment of uncomplicated SAM and MAM at 6 months post-randomisation and in terms of recovery rate in the treatment of uncomplicated SAM (WHZ < − 3 or MUAC < 115 mm or oedema) and also among children admitted with MUAC < 115 mm.

## Objectives

The principal objective aims to determine, 6 months after inclusion, whether the OptiMA strategy or ComPAS strategy leads to a favourable outcome which is non-inferior to what Niger standard protocol, in use in the same outpatient health facilities, provides. The definition of a favourable outcome is described in the “Study outcomes” section.

Two main secondary objectives for this trial are as follows. The first one is to determine whether the recovery rate of children with uncomplicated SAM according to the current WHO definition [14] (e.g. MUAC < 115 mm or WHZ < − 3 or bilateral oedema) managed under the simplified OptiMA or ComPAS strategies is non-inferior to that of the national standard protocol of Niger. The second one is to determine whether the recovery rate of children admitted with MUAC < 115 mm or oedema (+, ++) managed under the simplified OptiMA or ComPAS strategies is non-inferior to that of the national standard protocol of Niger.

Other secondary objectives include description and comparison between the three study arms of the following outcomes: cost-effectiveness analysis, RUTF consumption, relapse after nutritional recovery, non-response after outpatient follow-up, recovery, and success in children who present with both wasting and stunting at inclusion, and to describe the nutritional and clinical status of children hospitalised while enrolled in the study.

## Methods

This protocol follows the Standard Protocol Items: Recommendations for Interventional Trials (SPIRIT) guidelines see Additional file 1 [15].

### Trial design and study period

This study is a non-inferiority individually randomised controlled clinical trial conducted at the health centre level and in the community. Participants are randomly assigned to either the OptiMA or ComPAS strategy arms (intervention = OptiMA or ComPAS) or the national standard protocol arm (control = standard). Inclusions started on March 23, 2021, for a 9-month period with an expected end in December 2021. All participants are followed for a 6-month period post-randomisation, with an active phase expected to end in June 2022.

### Study setting

The trial took place in Mirriah district, Zinder region, in the south-east of Niger and was nested within a medical and nutritional emergency humanitarian project under ALIMA supervision since 2009. The Mirriah district is one of the most populated in Niger, with an estimated population of 700,000 in 2019 and approximately 20% of children aged under 5 years old. Zinder region is particularly affected by AM, with prevalences of SAM and MAM showing sharp increases in 2020 at rates estimated to be as high as 4.9% (95% CI 4.2–5.8) and 13.0% (95% CI 11.7–14.5), respectively [16].

During the trial preparation phase, we visited all health centres in the district to select four of these according to the following criteria: outpatient therapeutic programme (OTP) activities, population size covered, and logistic conditions. These four health centres cover 242 villages with an estimated population of 178,535. Overall, 82 (34%) villages are more than 10 km away from the health centre.

### Eligibility criteria

Children aged 6–59 months, living for more than 6 months in one of the four health areas, and meeting at least one of the three following AM criteria are eligible for inclusion in the trial: MUAC < 125 mm and/or bilateral pitting oedema (+/++) and/or WHZ < − 3. Children with a medical complication requiring hospitalisation; no appetite; oedema grade +++; with a known allergy to milk, peanuts, or RUTFs; with a chronic disease (such as sickle cell anaemia, trisomy 21, congenital heart disease, neurological disease); or children already enrolled in a malnutrition programme are excluded from the trial.

Two categories of children are included in the trial but not eligible for randomisation for ethical concerns. First, children with WHZ < − 3 and MUAC ≥ 125 mm without oedema are systematically included in the control arm, as they are not eligible for RUTF regarding the definition of wasting in neither OptiMA nor ComPAS strategies. Second, children with a sibling already enrolled in the trial are systematically allocated to the study arm of the index sibling. Ultimately, children with MUAC < 125 mm and/or bilateral oedema and with no sibling already included in the trial are individually randomised.

### Study outcomes

The primary outcome will be judged by a binary composite indicator. Children classified as “favourable outcome” fulfil all of the following criteria: alive, not acutely malnourished per the anthropometric definition applied at inclusion (MUAC > 125mm and WHZ > − 3, and no oedema) and not having an additional episode of AM throughout the 6-month observation period. This outcome is measured at 6 months after randomisation. All other children are classified as “unfavourable outcome”.

The main secondary outcome #1 will be determined among children who meet the current WHO definition of SAM at inclusion (MUAC < 115 mm or WHZ < − 3 or oedema +, ++). The main secondary outcome #2 will be determined among children admitted with a MUAC < 115mm or oedema (+, ++). For these two subgroups, recovery is defined in each arm by a 4-week minimum duration of treatment, an axillary temperature < 37.5 °C, and an absence of bipedal oedema and a MUAC ≥ 125 mm for two consecutive weeks. This outcome is measured throughout the 6 months post-randomisation, meaning a child will be considered as recovered if he reaches these criteria during the 6-month follow-up in outpatient or village visits. Additional secondary outcomes are listed in Table 1.

**Table 1:** Primary and secondary outcomes in the OptiMA Niger trial

	Measurement variable	Method of aggregation	Time point
**Primary**			
Favourable outcome	Composite: alive, not acutely malnourished per the definition applied at inclusion, and no additional episode of acute malnutrition throughout the 6-month observation period	Proportion	6 months post-inclusion
**Main secondary**			
Recovery	Composite: absence of bipedal oedema and a MUAC > 125 mm for two consecutive weeks, a 4-week minimum duration of RUTF treatment, and a good clinical health (without fever)	Proportion	Throughout the 6-month follow-up period
**Secondary**^a^			
Cost-effectiveness	Incremental cost-effectiveness ratio (ICER)	Ratio	Throughout the 6-month follow-up period
Consumption of RUTF per children with a favourable outcome	Sachets of RUTF consumed	Mean	6-month post-inclusion
Consumption of RUTF per recovered children	Sachets of RUTF consumed	Mean	End of RUTF treatment initiated at inclusion
Average daily weight gain in SAM children recovered	g/kg/day	Daily	End of RUTF treatment initiated at inclusion
Average daily MUAC gain in SAM children recovered	mm/day	Daily	End of RUTF treatment initiated at inclusion
Hospitalisation	Children with at least one episode of hospitalisation	Proportion	Throughout the 6-month follow-up period
Relapse to a new episode	Children with MUAC < 125 or WHZ < − 3 *Z*-score or oedema after RUTF treatment	Proportion	After recovering from the first episode of acute malnutrition
Non-response	Children who did not reach the recovery criteria	Proportion	16 weeks after inclusion
WaST	Children associated with severe and moderate stunting with acute malnutrition	Proportion	Throughout the 6-month follow-up period

^a^All the secondary outcomes will be analysed for the overall population (MUAC < 125 mm or WHZ < − 3 or oedema) and for the secondary populations (SAM WHO and MUAC < 115 mm or oedema)

### Sample size

We determined the sample size using an expecting success rate of 68% in each arm for the principal objective and a recovery rate of 82% and 74% for the main secondary objectives #1 and #2, respectively. These hypotheses were based on the results found in the pilot non-comparative cohort study implemented in the Mirriah district in 2019 (Personal communication, 2020). Recovery rates under OptiMA protocol were estimated at 91%, 70%, and 57% for children admitted with a MUAC between 115 and 124 mm and WHZ > − 3, those with SAM according to the WHO definition, and those with MUAC < 115 mm, respectively. In the OptiMA-Niger trial, recovery is defined throughout the 6-month follow-up which will increase these estimations. After having been discharged from this study, non-responders and a sample of recovered children were also seen 6 months after their exit in order to estimate a relapse rate. We estimated that children will be followed up on average 4 months after discharge with a relapse rate of 30%.

To demonstrate non-inferiority of the OptiMA or ComPAS strategies with standard protocol, we assumed a non-inferiority margin of 10%, the same as in other non-inferiority trials assessing simplified protocol [11, 13]. We reduced the test significant level (alpha) at 1.25 to consider the multiple testing [17], a power of 90% for the main objective and 80% for the 2 main secondary objectives were applied, and an inflation rate of 5% was used to account for unexploitable data. Calculations were performed using the SAS software (Proc Power TwoSampleFreq). For the principal objective, 568 children are required per arm, and 295 and 384 children per arm for the main secondary objectives #1 and #2, respectively.

### Randomisation

Randomisation was performed using specially developed software, containing lists prepared in advance by an independent statistician, and inaccessible to trial staff. The randomisation is done in blocks (block size is kept confidential) and stratified at the study site by the severity of AM according to the following criteria: MUAC < 115 mm or oedema, MUAC > 115 mm and WHZ < − 3, and MUAC between 115 and 124 and WHZ > − 3 without oedema. This stratification allows for the recruitment of comparable groups of SAM WHO, MUAC < 115 mm, and non-SAM children and for simultaneous randomisation at the four sites. Once the randomisation was decided by the investigator on the basis of the verification of the eligibility criteria, they interrogated this software, which assigned the code and the corresponding treatment. This software assigns a treatment arm by sequentially drawing from this list each time a randomisation procedure is completed. After the children were assigned to an arm by the software, the trial and clinic staff were informed of the assigned treatment and therefore became unblinded. Once the sample size for the primary objective will be reached, only SAM WHO and then MUAC < 115 mm or oedema children will continue to be enrolled and randomised until the sample size required for the two main secondary objectives will be attained.

### Blinding

Due to the nature of the intervention, this is an open-label trial; treatment are assigned blindly by the randomisation software, but the trial staff involved, including data analysts, were aware of the arm allocation for each patient. Caretakers and study personnel are aware of the study arm allocation for participating children due to the differences in anthropometric criteria and RUTF ration. On inclusion, the nurse research officer gives each trial participant’s caretaker a card specifying the nutritional strategy being followed by the child. Caretakers are asked to present this card in the event of an unplanned outpatient consultation or hospital admission during the study period. In this way, nurses or doctors at any health facility are able to identify the nutritional treatment being followed by the child.

### Children selection procedure and enrolment

Prior to enrolment, the research team composed of community health workers (CHWs), nurses, clinical trial technicians, clinical trial monitors, data mangers, and Ministry of Health (MoH) medical staff implicated in the study were trained on the trial protocol, standardised operating procedures (SOP), and the correct use of IT tools developed for data collection. Clinical trial technicians were specifically trained to enrol and randomise study participants and to monitor home visits and outpatient visits at the health centre. Due to the SARS-Cov-2 pandemic, all staff were also trained on barrier measures (wearing a mask, physical distancing, interviewing outside, washing hands).

Children are directly recruited when they presented for outpatient consultation in one of the four health centres selected in the trial. To ensure the data quality required in a randomised controlled trial and to cover the AM peak (July to October), we control the number of inclusions per day. A maximum threshold is fixed at 7 inclusions per day and per clinical trial technician. When more than 7 children are eligible per day in a health centre, a standard operating procedure to ensure a random selection of children is implemented. Mothers with their children are used to arriving early at the health centre. MoH staff in charge of the screening at admission give a numbered card to the mother if the child is eligible for the trial (anthropometric criteria, age, and place of residence). Once most of the mothers have arrived, the research field coordinator is informed by phone of the number of eligible children and proceeds to the randomised selection of children from numbered balls in a bag. The following data on age, sex, MUAC, WHZ, and distance between the village and the health centre are collected for all eligible children to check on the representativeness of the sample. Children randomly selected are proposed to participate to the trial, and the others do not participate in the trial and follow the standard protocol.

During the screening at health centres, a MoH staff supervised by a clinical trial technician collects the anthropometric (weight, height, MUAC, WHZ), demographic (age, sex), and clinical (oedema) data on eligible children. A standard operating procedure for assessing child anthropometry (weight, height, MUAC, and oedema) is observed at each site (see Additional file 2). Then, children undergo a medical consultation to determine whether they meet the inclusion criteria.

### Informed consent procedure

For each eligible and selected child, the caretaker is given a study information sheet explaining the study’s aims, treatment protocols, study duration, and frequency of clinic visits and community follow-up. On the consent form, participants will be asked if they agree to the use of their data should they choose to withdraw from the trial. Participants will also be asked for permission for the research team to share relevant data with people from the universities taking part in the research or from regulatory authorities, where relevant. This information is explained orally in Haoussa (local language). Caretakers who agreed to participate in the study indicate their consent by signing (signature or fingerprint) a written consent form. When caretakers are not able to read or write, an impartial witness oversaw the consent process and attest to the caretaker’s consent by signing the consent form on her/his behalf. All caretakers are informed about their right to withdraw from the study at any time without affecting the quality of medical care provided to their children. All medical care and nutritional treatment are provided free of charge, regardless of participation in the study. This trial does not involve collecting biological specimens for storage.

### Intervention

Table 2 summarises the difference in treatment eligibility, recovery definition, and exit criteria between the current national protocol in Niger, OptiMA, and ComPAS strategies. The RUTF ration per week according to standard, OptiMA, and ComPAS strategies are detailed in Additional file 3.

**Table 2:** Wasting definition, treatment products, calculation of dosage, and recovery definition in the Niger national, OptiMA, and ComPAS protocols

	National Niger protocol			OptiMA protocol			ComPAS protocol	
	SAM	MAM		Acute malnutrition			Acute malnutrition	
*Wasting definition*	MUAC < 115 mm or WHZ < − 3 or bipedal oedema	MUAC (115–124 mm) or − 3 < WHZ < − 2 and without bipedeal oedema		MUAC < 125 mm or bipedal oedema			MUAC < 125 mm or bipedal oedema	
				MUAC < 115 mm or bipedal oedema	MUAC 115–119 mm	MUAC 120–124 mm	MUAC < 115 mm or bipedal oedema	MUAC 115–124 mm
*Treatment product and quantity*	RUTF 150–200 kcal/kg/day	If aged 6–23 months: super cereal plus 200 g/day (~ 787 kcal/day) or RUSF, one 92 g sachet/day (500 kcal/day)	If aged 24–59 months: no supplementation	RUTF 170–200 (kcal/kg/day)	RUTF 125–190 (kcal/kg/day)	RUTF 50–166 (kcal/kg/day)	RUTF 1000 kcal/day (2 sachets/day)	RUTF 500 kcal/day (1 sachet/day)
*Calculation of dosage*	According to the weight	Fixed amount, regardless of weight or MUAC status	–	According to MUAC status and weight			According to MUAC status	
*Frequency*	Weekly	Weekly	No visit	Weekly	Weekly	Weekly	Weekly	Weekly
*Recovery definition*	• MUAC ≥ 125 mm AND WHZ ≥ − 2 for 2 consecutive weeks • No oedema for 14 days • 4 weeks of participation in the programme • Good clinical health (no fever)			• MUAC ≥ 125 mm for 2 consecutive weeks • No oedema for 2 consecutive weeks • 4 weeks of participation in the programme • Good clinical health (no fever)			• MUAC ≥ 125 mm for 2 consecutive weeks • No oedema for 2 consecutive weeks • 4 weeks of participation in the programme • Good clinical health (no fever)	

*ComPAS* Combined protocol for Acute Malnutrition Study, *MAM* moderate acute malnutrition, *MUAC* mid-upper arm circumference, *OptiMA* Optimising acute MAlnutrition, *RUSF* ready-to-use supplementary food, *RUTF* ready-to-use therapeutic food, *SAM* severe acute malnutrition, *WHZ* weight-for-height *Z*-score

In the control arm, children included with a MUAC < 115 mm and/or WHZ < − 3 and/or nutritional oedema are treated weekly with RUTF, according to the Niger national protocol dosage table based on the child’s weight. The Niger protocol provides a RUTF daily caloric intake of 150–200 kcal/kg/day during the entire treatment schedule until recovery in children with SAM. Children under 2 years old included with a MUAC between 115 and124 mm and WHZ > − 3 without bipedal oedema are treated weekly by one sachet of RUSF per day (500 kcal/day) during the course of treatment. However, children over 2 years old meeting the same moderate anthropometric criteria are not eligible for supplementation according to the national MAM programme.

The simplified strategies apply to AM children defined as MUAC < 125 mm or bipedal oedema (+, ++) without medical complications on admission and with a good or moderate appetite test.

In contrast to the weight-based RUTF ration in the national SAM programme, the OptiMA RUTF ration is calibrated to the child’s degree of wasting based on the combination of MUAC status and weight. Thus, more nutritional support is given to the most severely malnourished children and then gradually reduced as the child’s MUAC increased. Children with MUAC < 115 mm or oedema receive 175 kcal/kg per day of RUTF. Children with MUAC 115–119 mm, either at admission or during the course of treatment, receive 125 kcal/kg per day of RUTF, and children with MUAC ≥ 120 mm receive 75 kcal/kg per day of RUTF (with a minimum of one sachet/day) until discharge from the programme.

The ComPAS strategy is the most simplified protocol, not requiring a dosage table and proposing to gradually reduce the dosage according to the child’s MUAC status only during the outpatient follow-up as follows: 2 sachets/day of RUTF to children with MUAC < 115 mm or oedema and 1 sachet/day for children in the 115–124 mm MUAC zone.

For both simplified strategies, children with oedema receive the same RUTF ration as children with MUAC < 115 mm until oedema are resolved, at which point their ration is determined by MUAC and weight.

All other aspects of the standard nutrition protocol are similarly applied to all children in the three arms. All children undergo malaria rapid testing upon admission and at any point during their participation if clinical signs of malaria are detected. All children with positive malaria rapid diagnostic test are prescribed an artemisinin combination treatment. Amoxicillin 50–100 mg/kg/day is prescribed for 7 days to all SAM children. Seasonal chemoprevention (SCP) campaigns were organised by the MoH during the malaria pic (July to October). Albendazole or mebendazole is given to children who are at least 12 months old and did not get deworming in the previous 4 months. Children who did not have all the recommended vaccines up to date were vaccinated especially the measles vaccine. Children requiring higher-level treatment are referred to the inpatient facility in Mirriah or at Zinder Hospital. Hospitalisation criteria and treatment procedures follow the national recommendations in Niger.

### Follow-up

Table 3 summarises participants’ follow-up during the trial. All participants are monitored for a 6-month period post-randomisation. During the treatment phase, all children are followed once a week in outpatient consultations in each arm. On discharge from treatment, or immediately after inclusion for MAM children aged over 2 years in the standard arm, children are consulted once a month at home until the 6 months post-inclusion is completed. During village visits, a nurse research officer assisted by one or two CHWs monitored the anthropometric and clinical status of these children, referring any child who needed nutritional or medical care to either the primary healthcare facility or to the Mirriah hospital.

**Table 3:** Schedule of enrolment, interventions, and assessments, OptiMA Niger

	Admission	Outpatient follow-up	Home visits	6-month post randomisation
Timeline	d0	4–16 weeks^1^	2–5 months^2^	
Frequency	Day	Weekly	Monthly	
**Enrolment**				
Eligibility screening	x			
Individual consent	x			
Randomisation	x			
Socio-demographic data	x			
Economic data	x	x^3^		
Vaccination data	x	x^3^		
**RUTF supplementation**				
Standard	x	x		
OptiMA	x	x		
ComPAS	x	x		
**Assessments**				
Nutritional and medical assessment	x	x	x	x
Hospitalisation if required		x	x	x
Adverse event		x	x	x

^1^Minimum of 4 weeks of RUTF supplementation and a maximum of 16 weeks for the first episode of AM and if relapse

^2^After discharge from the outpatient visit or for children not eligible for supplementation (standard arm aged 24–59 months with a MUAC 115–124 mm and WHZ > − 3 and no oedema)

^3^At the discharge outpatient visit

Failure to reach recovery criteria is defined after 16 weeks in the programme, then children are discharged from OTF as non-responders and consulted once a month at home until they reach the 6-month post-inclusion. For each non-responder discharged with a MUAC < 115 mm, a medical committee meets to decide on the medical follow-up of the child in the trial. Defaulters are defined as a child absent for 3 consecutive visits. CHWs trace children who are absent to motivate caretakers to return to the health facilities. If caretaker refuse to return to the health centre, the child is followed up once a month at home until the 6-month post inclusion period. When caretakers refuse the follow-up at home, the exit status of children is declared “confirmed defaulter” when the vital status is known and “lost to follow-up” when the vital status is unknown.

Children are discharged from outpatient treatment according to the anthropometric exit criteria of their allocation arm. In standard protocol, children need to achieve a MUAC ≥ 125 mm and a WHZ ≥ − 2 without oedema for two consecutive weeks while in OptiMA and ComPAS strategies, children will be discharged if they reach a MUAC ≥ 125 mm without oedema for two consecutive weeks. For each arm, all children will have to participate a minimum of 4 weeks in the programme with good clinical health (absence of fever) to be discharged from the OTF.

### Women’s incentives

SAM and MAM programmes have been operational in the Zinder region for 15 years, and RUTF/RUSF are regularly found for sale in markets. During the OptiMA Mirriah pilot non-comparative cohort study, women refused to participate in the study to integrate the standard protocol and receive a maximum dose of RUTF/RUSF (personal communication, 2020). To ensure that the trial is understood, accepted, and implemented in accordance with local customs and practices, meetings to explain the trial eligibility and procedures were organised with community representatives and with nutrition actors. During the trial’s active phase, regular oral presentations are held at the local level. Neither monetary nor financial incentives to compensate for the loss of dose reduction were offered, but soaps were given to caretakers especially those randomised in intervention arms at inclusion, at the end of outpatient follow-up, and at the exit of the trial to increase their adherence to the trial.

### Data collection and management

Data are collected using the national programme individual outpatient cards that were modified to include specific clinical and follow-up data. Additional paper forms are also completed to collect demographic and economic data. Clinical trial technicians are based at each of the four health facilities to ensure the consent process, enrolment, randomisation, and dosage quantities according to the trial’s SOP. They also assess the accuracy of anthropometric measurements taken by the health facility staff and the medical visit by the nurses. Randomisation is realised using a specific offline tool developed especially for the study by our partner PACCI, for a branch for the French National Agency for Research on AIDS and Viral Hepatitis in Abidjan, Côte d’Ivoire. Data collected on paper forms are entered by data clerk agents based on each site and supervised by a data manager, using the RedCAP software from the offline tablet.

Monitoring is ensured by two clinical trial monitors under the responsibility of the research field coordinator and his deputy based in Zinder, in compliance with the recommendations of Good Clinical Practice. All data and any adverse events, including deaths and hospitalisations, are rigorously monitored weekly both on-site and remotely, according to the data monitoring plan. To ensure patient safety and data integrity, the methodology and coordinating centre continuously supervised data management activities, and the senior scientific project leader and investigators performed field visits on a regular basis.

### Statistical analysis

All statistical analyses will be performed using the RStudio Software (Boston, MA, USA) as specified by the data analysis plan published on ClinicalTrials.gov prior to performing any analysis. Statistical tests will be carried out bilaterally with an alpha risk of 5% and unilaterally with an alpha risk of 1.25% for the non-inferiority analysis. Qualitative variables will be described in terms of numbers and percentages and provided with CIs when relevant. If necessary, comparisons of qualitative variables will be made using *χ*^2^ or Fisher exact tests. Quantitative variables will be described in terms of mean, SD, and CI or median, range, and IQR. If necessary, comparisons of quantitative variables will be made using Student’s, Wilcoxon’s, or Kruskal-Wallis tests, depending on the distribution of the variable of interest. Time delay type variables will be described in terms of the incidence of occurrence, and the probability of occurrence over time, estimated by the Kaplan-Meier method. If necessary, probability comparisons will be made using log-rank tests, or proportional risk models, after verification of the assumption of proportionality of risks.

The main analysis of the primary and secondary outcomes will be intention-to-treat and per-protocol given that this is a non-inferiority trial. Participants included in the main analyses will be described according to the diagram defined by CONSORT (Consolidated Standards of Reporting Trials) recommendations [18]. The occurrence of the primary “favourable outcome” and the secondary main outcome “recovery” will be compared between the two randomisation arms. These two comparisons will be performed both by “intention-to-treat” basis and on a “per-protocol” basis. Non-inferiority analyses will be performed on these two outcomes. Only if non-inferiority for these two outcomes is demonstrated will the other secondary analyses will be performed and, if appropriate, as a superiority analysis. Superiority will be particularly sought for cost outcomes related to RUTF cost-efficiency.

### Safety

The trial is monitored monthly by an international steering committee and twice a year by a national steering committee where any adverse events are presented. We also planned a field visit by the national steering committee during the enrolment phase. The data safety and monitoring board (DSMB) for this trial did not request any interim analysis, but security analyses are planned after 500 and 1500 inclusions and will consist in comparing across study arms mortality, hospitalisation, and life-threatening events rates. The DSMB will meet at least twice during the recruitment phase to make recommendations following the security analysis. The ethics committees did not require any meetings during the active phase.

A child included in one of the two intervention arms could be removed from the study if (s)he experiences two consecutive episodes of > 5% weight loss during the course of treatment and is hospitalised once, or if (s)he is not SAM at inclusion but deteriorates to SAM during RUTF supplementation.

### Ethics and dissemination

The study is conducted in accordance with the Declaration of Helsinki. We obtained ethical approval with annual renewal from the National Ethics Committee in Niger (065/2020/CNERS) and from the Ethics Evaluation Committee of the Inserm in France (CD/EB 21-025). The final version of the protocol, version 2.0, dated December 15, 2020, is available for sharing upon request and included minor amendments to the protocol version 1.0 which was approved by these two ethics committees and did not require a new authorisation. All data are anonymised when entered into the database using unique identification numbers. We intend to disseminate the results regardless of positive or negative findings via peer-reviewed publications, conferences, and clinical networks targeting academics, policy-makers, clinicians, and caregivers.

## Discussion

The OptiMA Niger trial presents a unique opportunity to generate much-needed evidence on various RUTF dosing strategies using the most robust trial design by individually randomising acutely malnourished children. This trial monitors the outcomes for an extended period of 6 months post-randomisation to capture recovery and relapse in the main outcome. This is also the first trial planning to evaluate the impact of reduced dose on the most vulnerable children (MUAC < 115 mm or oedema).

Should the ComPAS strategy be shown to be non-inferior to the current national protocol or OptiMA, this would provide the evidence for developing a more efficient nutrition protocol that could be implemented throughout the health system pyramid, beginning with CHWs. If OptiMA and ComPAS will be non-inferior to national protocol with respect to recovery rate for children with MUAC < 115 mm, the sample size will be sufficient to test whether OptiMA is in fact superior to ComPAS. If non-inferiority of ComPAS and OptiMA versus national protocol will not be demonstrated in each objective, then dose reduction will not be advisable for these children. Should OptiMA prove to be superior to ComPAS for the recovery of children MUAC < 115 mm but non-inferior to the current protocol that would pave the way for nutrition programming that is more efficient than current protocols and implementable at the level of the health post where weighing scales are available. The results of this trial should be interpreted together with the cost-effectiveness analysis to support policy decisions aimed at improving the coverage and quality of treatment.

### Trial status

The final version of the protocol is version 2.0, dated December 15, 2020. Enrolment of the trial began on March 22, 2021, and is expected to be completed in December 2021. The active phase will end 6 months after the last recruitment in June 2022.

## Supplementary Information

**Additional file 1.** SPIRIT Checklist for Trials.

**Additional file 2.** Standard operating procedure for taking anthropometric measurements.

**Additional file 3.** RUTF and RUSF ration per week according to Standard protocol of Niger, OptiMA and ComPAS strategies.

## Data Availability

The datasets generated during this study will be available from the corresponding author on reasonable request.

## Abbreviations

ALIMA: Alliance for international medical action
AM: Acute malnutrition
ComPAS: Combined protocol for acute malnutrition study
DRC: Democratic Republic of the Congo
CHW: Community health workers
CMAM: Community-based management of acute malnutrition
CI: Confidence intervals
CONSORT: Consolidated standards of reporting trials
DSMB: Data safety and monitoring board
HAZ: Height-for-age *Z*-score
Inserm: National Institute for Health and Medical Research
IRC: International rescue committee
MAM: Moderate acute malnutrition
MoH: Ministry of health
MUAC: Mid-upper-arm circumference
OptiMA: Optimising treatment for acute malnutrition
OTP: Outpatient therapeutic programme
RUTF: Ready-to-use therapeutic food
RDT: Rapid diagnostic tests
SAM: Severe acute malnutrition
SD: Standard deviation
SOP: Standardised operating procedures
SPIRIT: Standard protocol items: recommendations for interventional trials
RCT: Randomised controlled trial
WHO: World Health Organization
WHZ: Weight-for-height *Z*-score
