# Supplementary material for: Simplifying and optimising management of acute malnutrition in children aged 6 to 59 months: study protocol for a 3 arms community-based individually randomised controlled trial in decentralised Niger
Source: Trials. 2022 Jan 28;23:89. doi: 10.1186/s13063-021-05955-6 (PMC8796195; doi:10.1186/s13063-021-05955-6)
Supplement: Supplementary file 3 — Additional file 3. RUTF and RUSF ration per week according to Standard protocol of Niger, OptiMA and ComPAS strategies. [file 13063_2021_5955_MOESM3_ESM.docx]

**Additional file 3: RUTF and RUSF ration per week according to Standard protocol of Niger, OptiMA and ComPAS strategies.**
